# Preparation of Low-Surface-Energy SSBR@FA Hybrid Fillers via Solution Mechanochemical Approach and Its Enhancement in Mechanical Strength on the Modified FA/SBR Composites

**DOI:** 10.3390/polym18030348

**Published:** 2026-01-28

**Authors:** Wei Gao, Jiangshan Zhao, Wei Qi, Zhaohui Huang, Guofeng Liu, Chuanqi Feng, Chao Sang, Xiujuan Wang, Xiaolei Zhang

**Affiliations:** 1 Shandong Provincial Key Laboratory of Monocrystalline Silicon Semiconductor Materials and Technology, Dezhou University, Dezhou 253023, China; zhaojshtxy@163.com (J.Z.); chq_feng@dzu.edu.cn (C.F.); 18810988921@163.com (C.S.); zhangxl@dzu.edu.cn (X.Z.); 2 College of Chemistry and Chemical Engineering, Experimental Management Center, Institute of Biophysics, Dezhou University, Dezhou 253023, China; 13305345667@189.cn (W.Q.); gfliu26@163.com (G.L.); 3 State Key Laboratory of Advanced Optical Polymer and Manufacturing Technology/Key Laboratory of Advanced Rubber Material, Ministry of Education, Qingdao University of Science and Technology, Qingdao 266042, China; wangxj@qust.edu.cn

**Keywords:** fly ash, ball milling-surface modification, rubber, mechanical properties

## Abstract

Owing to the substantial polarity difference and weak interfacial interaction, the large-scale application of fly ash (FA) in rubber materials still faces substantial challenges. To solve this issue, this study prepared a modified hybrid SSBR@FA filler through a solution mechanochemical reaction between solution-polymerized styrene-butadiene rubber (SSBR) and FA in a lab planetary ball mill. Fourier transform infrared spectroscopy (FTIR) and energy-dispersive spectroscopy (EDS) analyses demonstrated the in situ grafting-neutralization between the carboxyl in the SSBR chains and metal oxides in FA. Transmission electron microscopy (TEM) showed that surface-grafted SSBR formed a rubber-constrained layer on FA particle surfaces, which can reduce their surface energy and improve the wettability between FA and SBR matrix. Compared with the SBR vulcanizate, the mechanical properties, thermal conductivity, and flame-retardant properties of the SBR/SSBR@FA vulcanizates were obviously improved. This was because of the uniform distribution of FA and the improved interfacial interaction between FA and the rubber matrix. For example, the tensile strength, tear strength, and elongation at break increased by 66.3%, 52.9%, and 17.7%, respectively. This easy, efficient, and environmentally modified method for FA was expected offer a practical and creative solution for its application in rubber manufacturing.

## 1. Introduction

Although wind, solar, and biogas power generation are practicable alternative energy sources at present, coal-fired power factory still play a vital role in the production of electricity. It accounts for nearly 41% of global power output, and the figure is expected to rise to 44% by 2030 [1,2,3]. The coal-fired power factory will produce about one ton of fly ash (FA) as a byproduct [1] when every four tons of coal is burned. In China, the annual production of FA reaches hundreds of millions of tons, yet its utilization rate remains at only about 45% [4,5], leading to serious environmental problems. In order to promote the recycling use of FA, researchers have put it into use in the construction industry [6], oil extraction [7], agriculture, and so on [8]. In addition, FA has the advantages of low cost, low density, strong filling capability, smooth surface, and good processability [9,10,11]. It can serve as a filler in polymer materials, especially rubber-based composites, which is a sustainable development strategy.

In the micromorphology, FA’s spherical structure is similar to carbon black [12], which is made from silica and metal oxides. At the same time, FA shows a low agglomeration tendency [10]. As a non-synthetic filler, the low cost and zero carbon emissions [2] of FA make it an ideal substitute for reducing both the production cost and environmental influence of rubber-based composites [12]. The incorporation of raw FA into rubber matrices reduced curing time but damaged mechanical properties compared to silica-filled rubber composites [13,14,15,16]. This is mainly attributed to the low structural dimensionality, low specific surface area, and surface inertness of unmodified FA, which decreases the interfacial contact between polymer chains and FA and impairs surface wettability, thereby weakening their interfacial interaction [13]. To overcome this issue, surface treatment is an indispensable step. Garde et al. used acid-base modification to alter the surface morphology of FA, but the modified FA-filled polyisoprene still exhibited low bound rubber content and poor mechanical strength, even with the addition of Si69 [17]. This is due to the large particle size of FA and the absence of surface-reactive groups [10,18,19]. Based on the above analysis, the key factors such as particle size, morphology, and interfacial activity make a big difference in the reinforcing efficiency of FA.

In terms of the chemical composition, in addition to silica, FA also contains substantial amounts of metallic oxides such as CaO, Al_2_O_3_, and Fe_2_O_3_. Because of this compositional feature, Yang’s group proposed the concept of in situ grafting-neutralization, aiming to enhance the performance of FA-filled rubber composites [1,7,10,19]. Under heating conditions, an in situ carboxylate reaction facilitated the immobilization of a carboxylated nitrile butadiene rubber (XNBR) layer on FA surfaces, which enhanced the interaction of the interface. Therefore, compared to pure XNBR, the mechanical properties of the XNBR composite, which was filled with FA, were improved significantly [10]. The research team also fabricated composite materials by incorporating sorbic acid (SA) or tannic acid (TA) into non-carboxylated rubbers along with FA. One end of SA or TA reacted with metallic oxides in FA filler, and the other reacted with rubber chains, which strengthened interfacial attachment in composite materials [1,7,19]. Following the same principle, FA was subjected to carbonation through CO_2_ by another research team [20]. The incorporation of modified FA resulted in different levels of enhancement in the mechanical and flame-retardant performance of silicone rubber.

Another effective strategy for improving wettability between FA and polymer was to modify FA with a hydrophobic polymer. Bordoloi et al. engineered a surface-modified FA by depositing a hydrophobic polysulfide layer on FA [12]. There was excellent wettability between surface-modified FA and natural rubber. Other research teams have also reported similar hydrophobic coatings based on biopolymers for FA encapsulation, synthesized from waste sulfur and cooking oil [21].

As a commonly used matrix in rubber composites, the works on the SSBR modifying FA are seldom addressed in the literature. Our earlier research introduced an easy, highly efficient, and environmentally modification method for preparing SSBR-grafted silica via solution mechanochemical processing in a lab-scale planetary ball mill [22]. The surface modification of silica reduced filler–filler interactions while enhancing the rubber-silica interfacial adhesion, and the performance of the SiO_2_-*g*-SSBR-filled SSBR composites was obviously improved. Inspired by these interesting results, two types of carboxyl-functionalized rubbers were subjected to wet ball milling with FA, aiming to prepare hydrophobic FA (SSBR@FA) through in situ grafting-neutralization in this work. Ball milling was employed to decrease the particle size of FA and alter the structure of FA [2]. Under the dual effects of ball milling and SSBR modifiers, the mechanical properties, thermal conductivity, and flame retardancy of SSBR@FA-filled SBR vulcanizates have been improved, offering a practical and transformative solution for industrial-scale application of FA in rubber manufacturing.

## 2. Materials and Methods

### 2.1. Materials

FA (BET surface area of 3.38 m^2^/g) was provided by a thermal power plant, and the composition is depicted in Table S1. SSBR (*M*_n_ = 74,300 g/mol, PDI = 1.03), with 32.4 wt% styrene and 47.9 wt% 1,2-polybutadiene units relative to polybutadiene, was supplied by Zhejiang Zhongli Synthetic Materials Technology Co., Ltd. (Jiaxing, China). 3-mercaptopropionic acid (MPA), 11-mercaptoundecanoic acid (MUA), and dilauroyl peroxide (LPO) (≥98%) were purchased from Adamas (Shanghai, China) and used in their original form. SBR1502 was purchased from Sinopec Qilu Petrochemical Company (Zibo, China). Other reactants, such as cyclohexane, zinc oxide, stearic acid, N-cyclohexylbenzothiazole-2-sulphenamide, 2-Mercaptobenzothiazole, and sulfur, were commercially sourced.

### 2.2. SSBR Functionalization

The procedure for carboxyl functionalization of SSBR through click reaction was explained in detail below. Briefly, under a nitrogen atmosphere, a predetermined amount of SSBR and cyclohexane were sequentially added to a glass reaction flask. The mixture underwent continuous stirring at 60 °C over a 6 h period until complete dissolution of SSBR in the cyclohexane, obtaining a homogeneous solution with a mass fraction of 10%. The SSBR solution subsequently received an injection of precisely measured thiol, either MPA or MUA. The mixture was heated at a temperature of 80 °C with simultaneous stirring for 10 min, and then a pre-prepared solution of LPO in cyclohexane was added to the mixture. The thiol-ene click reaction was maintained at 80 °C for 1 h under stirring conditions. The procedure for the post-treatment of carboxyl-functionalized SSBRs was similar to that of our previous work [22,23]. The final modified products were named as SSBR-*g*-MPA and SSBR-*g*-MUA, respectively.

### 2.3. Preparation of SSBR@FA

A Qm-2 laboratory planetary ball mill (Beijing New Lasers Technology Co., Ltd., Beijing, China) was employed to fabricate modified FA (SSBR@FA) with varying carboxyl-functionalized SSBR contents. The mill pot was sequentially charged with a cyclohexane solution of carboxyl-functionalized SSBR and FA at mass ratios of 0.01% and 0.03% (rubber to FA). A suitable amount of cyclohexane was then added to the mill pot to maintain a fly ash concentration of 40%. The FA and carboxyl-functionalized SSBR underwent grinding for 8 h with alternating clockwise and counterclockwise rotation. The planetary ball mill was operated at 260 rpm (revolution speed) and 530 rpm (rotation speed), using 30 zirconia balls (5 mm in diameter) and 18 zirconia balls (10 mm in diameter). To measure the typical structure of SSBR@FA, an appropriate amount of ball-milled FA was extracted using cyclohexane for 72 h to eliminate ungrafted SSBR. The purified SSBR@FA was dried in a vacuum drying oven at 50 °C for 36 h. The modified FA samples were named 1PFA, 3PFA, 1UFA, and 3UFA (e.g., 1PFA and 1UFA indicate that the mass ratio of SSBR-*g*-MPA and SSBR-*g*-MUA to FA was 1%, respectively).

### 2.4. Fabrication of SBR-Based Composites

SBR-based composites were fabricated by using traditional methods on a two-roll mill. The formulation for FA-filled SBR-based composites is shown in Table 1. The obtained composites filled by varied proportions of SSBR@FA were designated as follows: SBR/1PFA-15, SBR/1PFA-30, SBR/3PFA-15, SBR/3PFA-30, SBR/1UFA-15, SBR/1UFA-30, SBR/3UFA-15, and SBR/3UFA-30 (for example, SBR/1PFA-15 denotes a 15% mass ratio of 1PFA to SBR1502). As a contrast, SBR vulcanizate and SBR composites with raw FA were also prepared according to similar formulations, and the final products were termed as SBR, SBR/FA-15, and SBR/FA-30.

### 2.5. Testing and Characterization Methods

The Fourier transform infrared spectroscopy (FTIR) analysis of SSBR and FA was conducted by a Nicolet iS20 spectrometer (Thermo Fisher Scientific, Waltham, MA, USA). ^1^H nuclear magnetic resonance (^1^H NMR) was conducted with a Bruker Plus spectrometer (Bruker Corporation, Karlsruhe, Germany) (600 MHz) to analyze the microstructure of SSBR using CDCl_3_ as the solvent. The contact angles of FA with water and diiodomethane were determined by the SZ-CAMC32 equipment (Shanghai Xuanzhun Instrument Co., Ltd., Shanghai, China), and the surface energy was calculated via the Owens–Wendt equation. The thermal properties of FA were measured through thermal gravimetric analysis (TGA) with TGA/DSC3+ instruments (METTLER TOLEDO, Greifensee, Switzerland) from 30 to 800 °C. The temperature was increased at a rate of 10 °C/min. The specific surface area (SSA) of FA was determined using the BET method with an automated surface area and pore size analyzer (Quantachrome Nova 4000e, Boynton Beach, FL, USA) under nitrogen adsorption conditions. Prior to testing, the FA underwent degassing at 120 °C for 12 h. The surface morphology and elemental mapping of SSBR@FA and SBR/SSBR@FA vulcanizate were conducted by a scanning electron microscopy (SEM, TESCAN MIRA LMS, Brno, Czech Republic) equipped with energy-dispersive spectroscopy. The experimental cross-section of the SBR/SSBR@FA vulcanizate was obtained by fracturing after cryogenic embrittlement in liquid nitrogen. The morphology of the SSBR@FA was observed by using a transmission electron microscopy (TEM; H-800, Hitachi High-Technologies Corp., Tokyo, Japan). The mechanical properties of the vulcanizates were measured through a CMT4104 electronic tensile tester (Shenzhen SANS Test Machine Co., Ltd., Shenzhen, China) in accordance with ASTM D638 standards [24]. with a crosshead speed of 500 mm/min. The FA network was analyzed using an RPA 2000 instrument from (Alpha, CA, USA) at 60 °C. For the SBR/FA compounds, the strain range was 0.28% to 200% at a frequency of 1 Hz. For the SBR/FA vulcanizate, the strain range was 0.28% to 41.99% at a frequency of 10 Hz. To elucidate the viscoelastic properties of the SBR/FA vulcanizate, a VA3000 DMA (Metravib Corporation, Paris, France) was employed at 10 Hz in tension mode, heating at 3 °C/min from −60 to 90 °C. The strain amplitude of the test was 0.1%. The thermal conduction of the SBR/FA vulcanizate was assessed by the Hot Disk method TPS2500S (Hot Disk, Gothenburg, Sweden). The flame-retardant property was evaluated according to ISO4589 [25] via a JF-3 oxygen index apparatus (Zhide Innovation Instrument Equipment Co., Ltd., Beijing, China).

## 3. Results and Discussion

### 3.1. Synthesis of the Functionalized SSBRs

**Figure 1:**
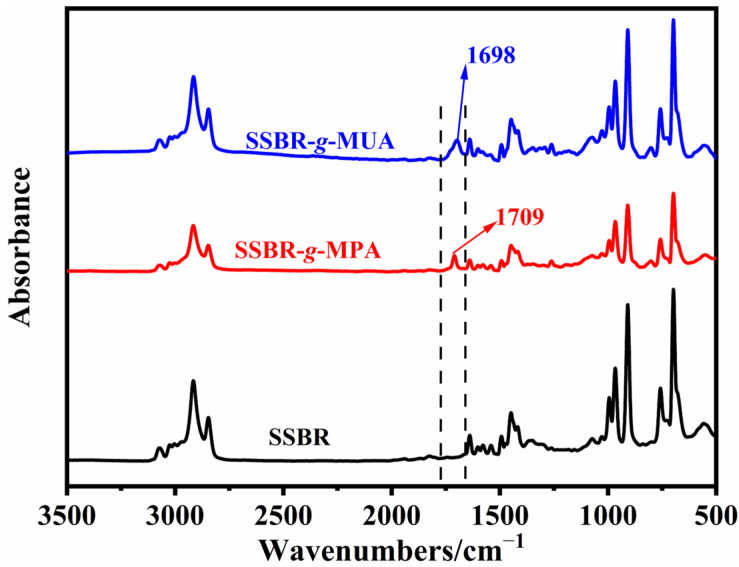
FTIR spectra of SSBR-*g*-MUA, SSBR-*g*-MPA, and SSBR.

To verify the click chemistry reaction between either MPA or MUA and SSBR, Figure 1 illustrates the FTIR spectra of pure SSBR and modified SSBR. It can be observed clearly that the peaks at 1709 cm^−1^ in SSBR-*g*-MPA and 1698 cm^−1^ in SSBR-*g*-MUA were assigned to the stretching vibrations of carboxyl moieties in hydrogen-bonded acid dimmer states, respectively [10,26]. The alkyl chain length of MUA was longer, and the carboxyl groups were positioned farther from the main chain of SSBR, thereby reducing the number of carboxyl groups embedded within the rubber matrix. This increased the probability of hydrogen–bonding and imposed greater constraints on the carboxyl groups. Compared with SSBR-*g*-MPA, the carboxyl groups’ vibrational absorption peak in SSBR-*g*-MUA exhibited a red shift of 11 cm^−1^ due to these structural differences.

**Figure 2:**
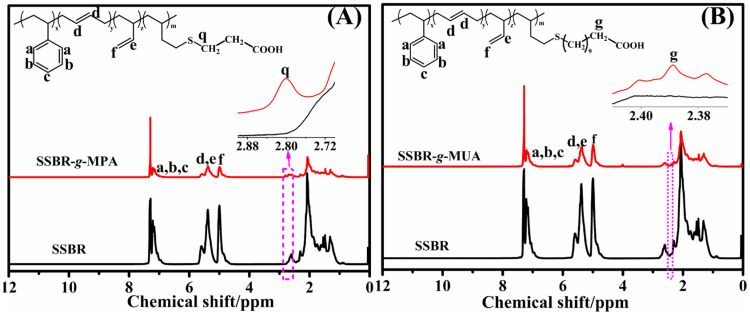
^1^H NMR spectra of (**A**) SSBR and SSBR-*g*-MPA and (**B**) SSBR and SSBR-*g*-MUA.

Figure 2 displays the ^1^H NMR result of rubber in this work. It should be noted that thiol-ene click chemistry predominantly occurred between 1,2-polybutadiene units (1,2-PB) and thiols [22,23,27,28,29]. Consequently, the content of 1,2-PB should decrease if the thiol-ene click occurred. According to the ^1^H NMR spectra, a new characteristic peak at 2.76–2.88 ppm in SSBR-*g*-MPA was attributed to the methylene protons from -SCH_2_CH_2_COOH, and a new peak at 2.37–2.41 ppm stemmed from methylene protons (-CH_2_COOH) in SSBR-*g*-MUA, respectively [23], which demonstrated that MPA and MUA were successfully grafted onto the SSBR chain. Furthermore, on the basis of the established equations in our previous work [23], the mass fraction of 1,2-PB (relative to PB) in SSBR decreased from 47.9% to 44.9% in SSBR-*g*-MPA and 47.3 in SSBR-*g*-MUA, which means that the grafting percentages of MPA and MUA were 6.3% and 4.7%, respectively.

### 3.2. FA Modification Through Ball-Milling-Induced Grafting of Carboxyl-SSBR

**Figure 3:**
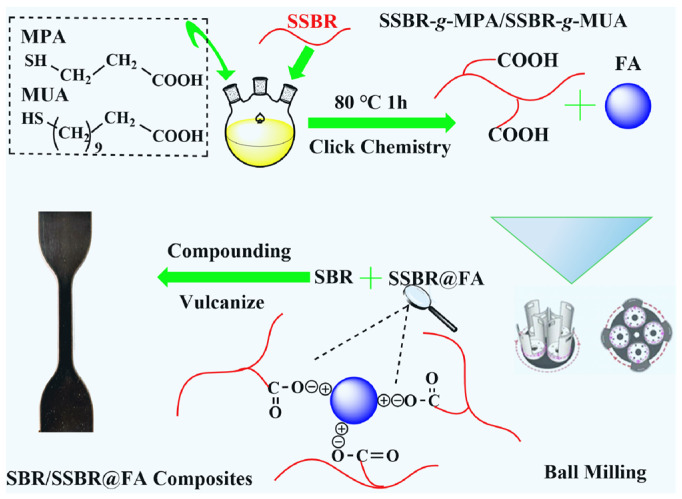
The design schematic of SBR/SSBR@FA composites.

It is well known that the carboxyl group can undergo a chemical reaction with metal oxides on the surface of FA and form carboxyl–metal bonds [1,7,10,19] under heating conditions. A planetary ball mill generates significant friction and impact forces [30] in the process of grinding. This provides higher energy input and chemical reaction rates [31,32,33] in comparison to the thermal-induced chemical reaction, and enables the mechanochemical activation at a lower temperature [34,35,36]. Therefore, carboxyl-metal chemical reactions are theoretically feasible under ball-milling conditions. Figure 3 presents the modification mechanism of FA.

**Figure 4:**
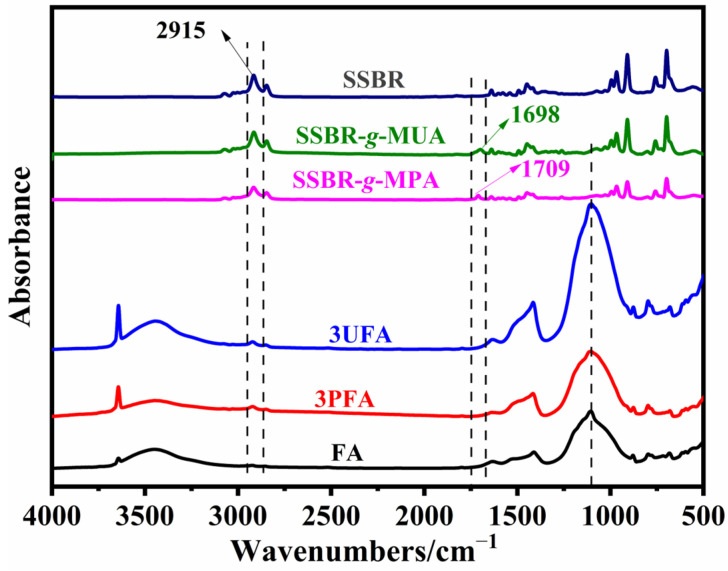
FTIR spectra of SSBR, functionalized SSBR, FA, and SSBR@FA after extraction.

It is reported that infrared spectroscopy exhibits high sensitivity to dipole moment changes in functional groups, making it a key analytical tool for investigating carboxyl-metal chemical reactions [1,7,10,19,26]. Figure 4 presents the corresponding spectrum of the SSBR@FA hybrid filler after extraction. After ball milling with FA, the disappearance of the stretching vibrations of carboxyl groups in hydrogen–bonded acid dimer states of two functionalized SSBRs indicated that the carboxyl groups underwent chemical reactions. The characteristic peaks of Si-O-Si in 3PFA and 3UFA became sharper, and the characteristic peak of Si-O-Si in 3UFA exhibited a slightly red shift (from 1105 cm^−1^ to 1103 cm^−1^) compared to those of FA. The Si-O-Si groups on the FA surface, interacting with the carboxyl–metal bonding groups, experienced restricted vibrational motion [19,37,38,39]. In addition, the characteristic peak at 2915 cm^−1^ was attributed to the stretching vibration of methylene groups in SSBR [22]. Notably, this characteristic peak remained observable in the FTIR spectra of the extracted 3PFA and 3UFA samples. In conclusion, under the action of ball milling, the functionalized SSBRs were grafted onto the surface of FA. Similar experiment results also could be found anywhere [22].

**Figure 5:**
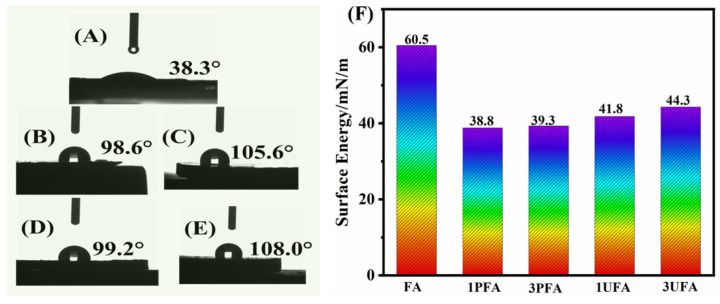
Water contact angle on the surface of (**A**) FA, (**B**) 1PFA, (**C**) 3PFA, (**D**) 1UFA, and (**E**) 3UFA. (**F**) Surface energy values of FA, 1PFA, 3PFA, 1UFA, and 3UFA.

The significant polarity difference between rubber and fillers leads to poor wettability, which not only hinders the dispersion of filler particles but also limits the enhancement of the composite composite’s overall performance [10,22,23,40]. The static contact angle measurement was used to investigate the polarity changes in FA. As Figure 5A displays, it is clear that water spread quickly on the raw FA surface with a water contact angle (WCA) of 38.3°, which shows an obvious hydrophilic nature of FA. After the FA was treated with SSBR, the water displayed spherical droplets formed on the modified FA surface with a WCA consistently exceeding 90°. SSBR@FA showed a pronounced hydrophobicity and an obvious change in surface properties. The surface energy of FA was calculated using the Owens–Wendt equation by determining its contact angles with water and diiodomethane. The concrete values of FA, 1PFA, 3PFA, 1UFA, and 3UFA are displayed in Figure 5F. It showed that the surface energy of modified FA was significantly reduced. Compared with the raw FA (60.5 mN/m), the surface energy of 1PFA decreased remarkably by 35.9%. The reduction in surface energy contributed to the improvement of wetting between FA and rubber, thereby enhancing the interfacial interaction of the SSBR@FA hybrid filler with the SBR rubber matrix. To visually demonstrate the hydrophobicity of modified FA, we immersed raw FA, ball-mill-treated FA (BFA) without carboxyl functionalized SSBR, 3PFA, and 3UFA samples in cyclohexane (Figure S1). Initially, all samples were well-dispersed in the cyclohexane and formed a turbid suspension. After ten minutes, the raw FA and BFA gradually settled to the bottom of the container, leaving a transparent supernatant. While the 3PFA and 3UFA remained partially dispersed, maintaining a turbid appearance in the solution.

**Figure 6:**
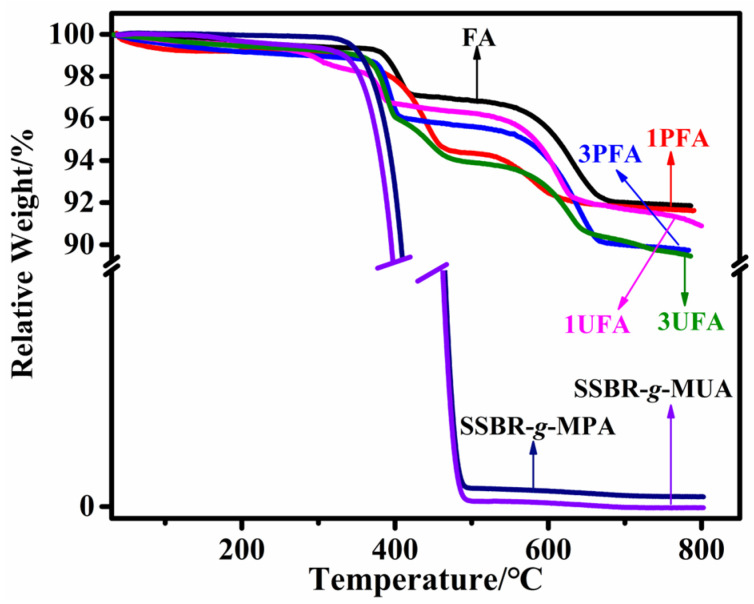
TGA curves of FA, functionalized SSBR, 1PFA, 3PFA, 1UFA, and 3UFA after extraction.

Figure 6 displays the thermogravimetric curves for FA, SSBR@FA, SSBR-*g*-MPA, and SSBR-*g*-MUA. The TGA curve of FA exhibited three weight loss regions. In the initial region (40–210 °C), the weight loss resulted from the removal of the unbonded water evaporation located on the surface [41,42]. The second region (210–450 °C) was assigned to the desorption of hydration water and devolatilization [42]. The final stage (450–780 °C) was due to the decomposition of residual coal in the FA [43]. The functionalized SSBRs were primarily decomposed within the temperature range of 220–490 °C. The TGA curve of SSBR@FA showed a notable weight loss above 300 °C, corresponding to the decomposition of functionalized SSBR [21]. According to further calculations, the grafting percentages of modified SSBR based on FA observed for 1PFA, 3PFA, 1UFA, and 3UFA were 0.2%, 2.1%, 0.7%, and 2.4%, respectively. Even after 72 h of extraction, the SSBR was not entirely removed, confirming that the SSBR was firmly adsorbed onto the FA surface through chemical bonding [44]. Moreover, the grafting efficiency of SSBR-*g*-MUA based on FA was higher than that of SSBR-*g*-MPA.

**Figure 7:**
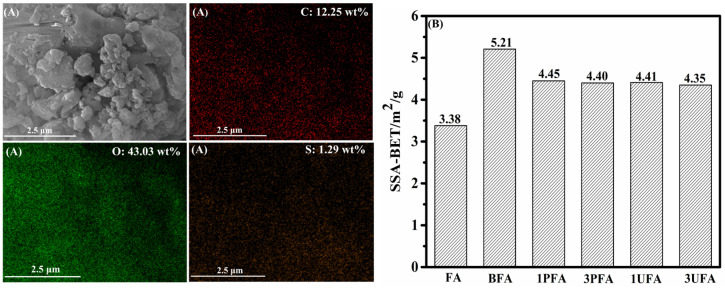
(**A**) SEM and EDS mapping diagrams of 3PFA and (**B**) SSA-BET values of FA, BFA, and SSBR@FA.

The SSBR molecular chains grafted onto the FA surface can be further confirmed by the chemical composition obtained from EDS. The EDS spectra of 3PFA and FA are shown in Figure 7A and Figure S2, respectively. The percentages of C, O, and S elements in 3PFA were all higher than those in FA, indicating that a SSBR layer was successfully coated on the FA. Furthermore, the SSAs of raw FA and SSBR@FA were determined by the BET method, and the corresponding data are presented in Figure 7B. Since ball milling can reduce FA particle size and change its morphology, the SSA of ball-mill-treated FA (BFA) without carboxyl functionalized SSBR was also measured. After ball milling, the SSA of BFA increased from 3.38 m^2^/g to 5.21 m^2^/g, representing a 54.1% increase compared to FA. While the SSAs of PFA and UFA were reduced compared to BFA, owing to the SSBR preventing nitrogen from accessing the pore area of FA [22,45,46]. However, the SSAs of SSBR@FA were still higher than those of raw FA. This promoted easier penetration of the rubber matrix into the pores of SSBR@FA and strengthened the interfacial bonding between the SSBR@FA hybrid filler and the rubber matrix. This was in agreement with Liu’s work, which reported the surface modification of FA using waste engine oil under mechanical activation [44]. Figure S3 shows the TEM results of 1PFA and 3UFA. It was evident that SSBR was adsorbed on the FA surface, and the SSBR@FA exhibited a core–shell structure, which enhanced the wettability between FA and SBR matrix.

### 3.3. FA-Reinforced SBR Composites

**Figure 8:**
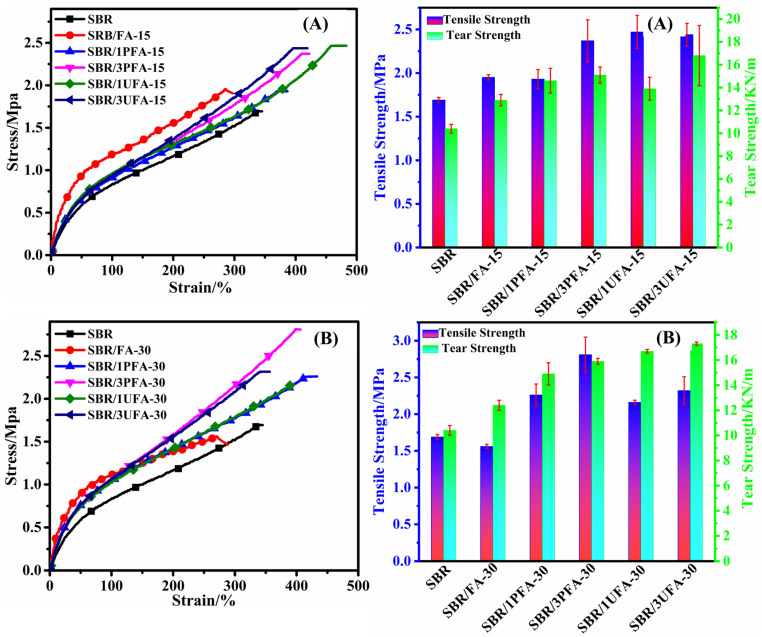
Stress–strain curves and mechanical properties of pure SBR, SBR/FA, and SBR/SSBR@FA vulcanizates with (**A**) 15 phr filler and (**B**) 30 phr filler.

Figure 8 shows the mechanical properties of all vulcanizates. According to the summarized results in Table 2, the addition of raw FA (15 phr) in SBR enhanced the tensile strength from 1.69 MPa to 1.95 MPa in comparison to pure vulcanized SBR, while the elongation at break was reduced from 345% to 284%. When the content of FA increased to 30 phr, the mechanical properties of SBR/FA further decreased, which demonstrated that the raw FA provided no reinforcement.

In contrast, the tensile strength, tear strength, and elongation at break of SBR/SSBR@FA vulcanizates were improved simultaneously, indicating that FA grafted with SSBR molecular chains not only served as a reinforcing filler but also further enhanced the elastic property of rubber-based composites in this system. This was in accordance with other work reporting the effects of filler modification on the properties of elastomeric composites [47]. For instance, the tensile strength, tear strength, and elongation at break of the SBR/3PFA-30 vulcanizate increased by 66.3%, 52.9%, and 17.7%, respectively, compared with those of the SBR vulcanizate. More interestingly, though adding the SSBR@FA into the SBR matrix, the modulus at 100% strain remained virtually unchanged, indicating that the dominant variable in the low-strain region is primarily determined by the changes in the bond angles of the rubber molecular chains [7,19]. Compared to pure SBR, the modulus of SBR/SSBR@FA in the 300% high-strain region increased, and this enhancement became more pronounced with higher SSBR@FA loading. This indicated that the SSBR@FA acted as a reinforcing filler.

**Figure 9:**
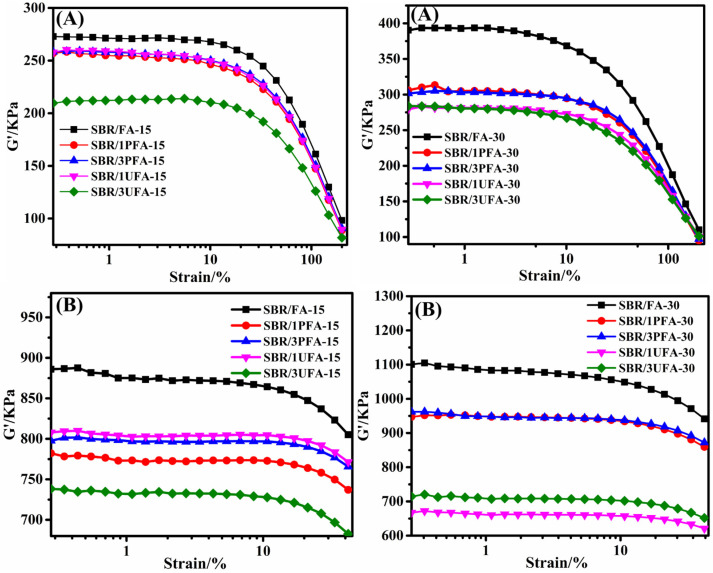
Payne effect study of SBR/FA and SBR/SSBR@FA composites: (**A**) compounds and (**B**) vulcanizates.

The well-known Payne effect demonstrates that the storage modulus (G’) of composites declines as strain increases [48]. The RPA (Rubber Process Analyzer) testing method was employed to investigate the impact of ball-mill grafting modification on the filler-filler network and dispersion of FA fillers. The resulting curves of G’ versus strain are displayed in Figure 9, with the corresponding results presented in Table S2. In this study, the SBR/FA compounds and vulcanizates displayed the highest initial G′ value and most significant Payne effect, suggesting a robust filler–filler network structure formed due to weak SBR-FA interfacial interaction. After chemical grafting of functionalized SSBR onto FA surfaces, the initial G′ values of SBR/SSBR@FA composites decreased with a reduction in the Payne effect. This was because the FA–FA networks were damaged considerably after SSBR was chemically grafted onto the FA surface. For instance, the initial G′ values of the SBR/1UFA-30 compound and vulcanizate decreased by 28.4% and 39.4%, respectively. As depicted in Figure 9, compared to SBR/FA, the G′ values at which filler–filler structures began to break in SBR/SSBR@FA vulcanizates increased. This indicated that the strain dependence of the storage modulus is reduced, which further demonstrated that the FA-FA filler network structure was damaged after grafting modification [23].

**Figure 10:**
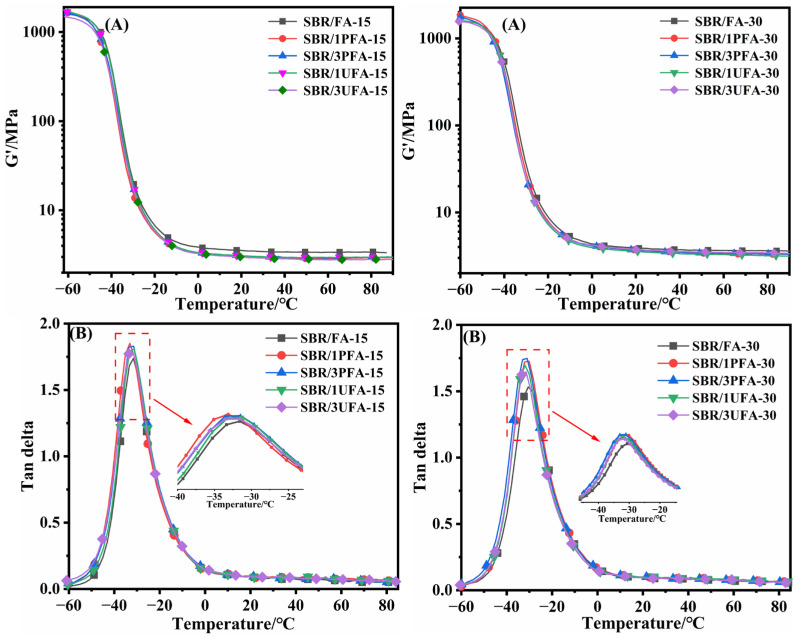
(**A**) G′-temperature curves and (**B**) loss factor–temperature curves of SBR/FA and SBR/SSBR@FA vulcanizates.

**Figure 11:**
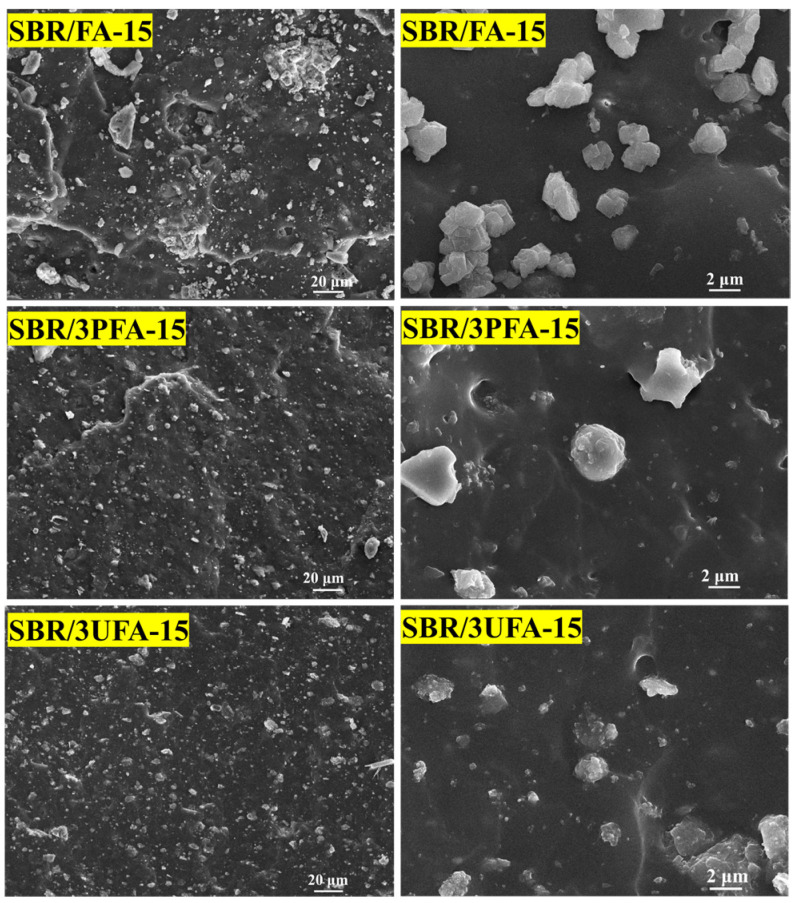
SEM images of SBR/FA-15, SBR/3PFA-15, and SBR/3UFA-15 with different magnifications.

The viscoelastic properties of the vulcanized rubber are displayed in Figure 10. The G′ values of vulcanized rubber in the rubbery plateau region were reported to reflect the filler–filler network [22,23,46,49]. A higher storage modulus value in the rubbery plateau indicates a stronger filler–filler network structure and poorer filler dispersion. The G’ values of the SBR/SSBR@FA vulcanizate, either filled with 15 phr SSBR@FA or 30 phr SSBR@FA in the rubbery plateau, are lower than those of the corresponding SBR/FA (Figure 10A). This indicated that the ball-mill-grafted modified FA reduced the filler–filler network structure and improved the degree of dispersion in the SBR matrix. The results were consistent with the subsequent SEM results (Figure 11). As Figure 10B displays, the tan *δ* values at *T*g (tan *δ*_max_) of SBR/SSBR@FA vulcanizates were higher than those of corresponding SBR/FA, implying stronger interactions between SBR and SSBR@FA [1]. This result can be explained as follows: compared with FA, SSBR@FA demonstrated a higher SSA and lower surface energy. These properties increased the contact area and wettability between SSBR@FA and SBR and enhanced their interfacial interaction.

Figure 11 presents the SEM images of SBR/FA-15, SBR/3PFA-15, and SBR/3UFA-15. The SEM micrographs of the cross-section of the SBR/FA composite after liquid nitrogen embrittlement fracture showed a multilamellar structure with concave holes. Furthermore, the micrograph showed obvious FA agglomeration, which had a bad influence on the composite’s mechanical properties. The majority of FA were predominantly distributed on the SBR matrix surface, which indicated poor wetting behavior with minimized interfacial contact. The cross-section of the SBR/SSBR@FA composites showed reduced multilamellar structures and concave pits and presented a comparatively featureless fracture surface. Compared to SBR/FA, they showed a clearly distinct morphology. Moreover, SSBR@FA particles were embedded within the SBR matrix, which exhibited reduced interfacial chromatic contrast. This enhanced compatibility was a result of the dual effects of ball milling and SSBR modification, which increased FAs specific surface area and decreased its surface energy at the same time, thereby optimizing interfacial contact area and wettability with the rubber matrix [2,22]. This observation was consistent with the previously presented BET specific surface area and surface energy results.

It is well known that the mechanical properties of rubber composites are decided by a combination of factors, which include the filler’s morphology and structure, the interfacial adhesion between rubber and filler, as well as the chemical crosslinking and confinement effects [47,50,51,52,53]. In this study, both ball milling and grafting can influence the aforementioned factors. Firstly, the intense friction and impact forces that were generated during the grinding process roughened the FA surface and increased its specific surface area. This increased in the contact area and mechanical adhesion between FA and the SBR matrix [47]. Secondly, the SSBR molecular chains grafted onto the FA surface through solution mechanochemistry and formed a rubber-constrained layer around the FA particles [10]. Also, grafted SSBR acted as a bridge between SBR and FA and further enhanced the interfacial interaction between them. Thirdly, the microscopic network structure formed by physical entanglement between grafted SSBR and SBR matrix enhanced constraint and improved the mechanical properties [1,7,10,19]. When the SBR/FA composite was subjected to external force, stress spread along the macromolecular chains of the rubber to prevent stress concentration. Due to the increased contact area between SSBR@FA and the SBR matrix [2,20], the interfacial interaction was enhanced. As a result, the probability of SSBR@FA particle de-bondment at the interface was reduced. This made the transfer of external forces through the interface [22,54] more efficient and extended the propagation paths of tear cracks [22,49]. Thus, SBR/SSBR@FA could withstand greater external forces and exhibited improved mechanical properties.

**Figure 12:**
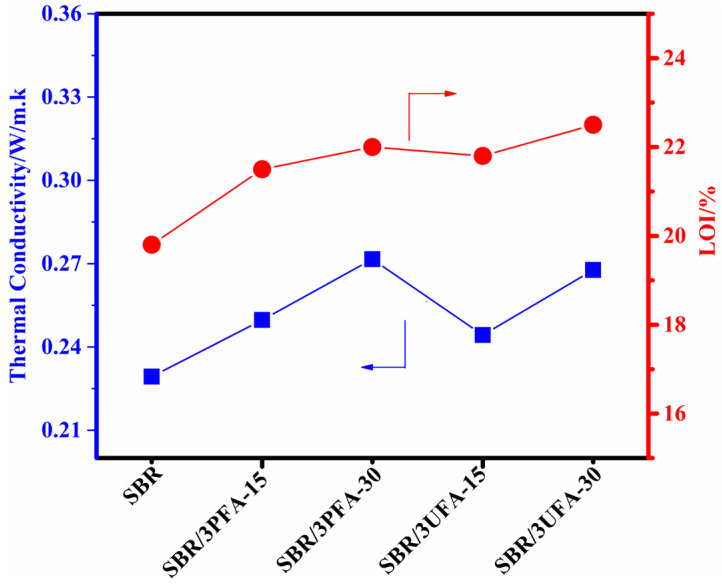
Thermal conductivity and LOI of SBR/FA and SBR/SSBR@FA vulcanizates.

The thermal conductivity and flame-retardant properties of SBR/FA and SBR/SSBR@FA composites were investigated by measuring the thermal conductivity and limiting oxygen index (LOI). The corresponding data are provided in Figure 12 and Table S3. Compared with pure SBR, the thermal conductivity values of SBR/3PFA-15, SBR/3PFA-30, SBR/3UFA-15, and SBR/3UFA-30 increased by 8.9%, 18.4%, 6.5%, and 16.7%, respectively. Meanwhile, the LOI values of SBR/3PFA-15, SBR/3PFA-30, SBR/3UFA-15, and SBR/3UFA-30 increased by 8.6%, 11.1%, 10.1%, and 13.6%, respectively. This enhancement can be attributed to the improved dispersion of FA in composites achieved through ball-mill graft modification, which effectively leveraged the heat-conduction [55,56] and flame-retardant effects of Al_2_O_3_ and SiO_2_ in FA. Additionally, the increased SSA of SSBR@FA facilitated the distribution of CO2 generated during combustion within the pores on the surface of the FA, which created an oxygen barrier effect and further improved the flame-retardancy.

## 4. Conclusions

The mechanochemical activation obtained through ball milling facilitated an in situ carboxylate reaction between carboxyl functional groups in SSBR and metal oxides on the FA surface. The results of FTIR confirmed the successful grafting of SSBR-*g*-MPA and SSBR-*g*-MUA onto FA, with maximum grafting percentages of 2.1% and 2.4%, respectively. Under the combined effects of ball milling and grafting, the modified FA exhibited hydrophobicity, an increase in specific surface area, and a reduction in surface energy. The SSBR@FA exhibited enhanced wettability with the SBR matrix, as confirmed by SEM results, as a result of the dual role of SSBR-*g*-MPA/SSBR-*g*-MUA, which served as both a dispersant and rubber matrix. With the enhanced interfacial interaction and a rubber-constrained layer surrounding the FA particles, external forces can be transferred at the interface more efficiently. As a result, the mechanical properties of the composites were enhanced. Even when 30 phr of SSBR@FA was added, the tensile strength, tear strength, and elongation at break increased by 66.3%, 52.9%, and 17.7%, respectively, compared with those of pure SBR vulcanizate. The incorporation of SSBR@FA also led to an enhancement in both the thermal conductivity and flame resistance of the vulcanizates. For example, compared with those of pure SBR vulcanizate, the thermal conductivity and LOI values of SBR/3PFA-30 vulcanizate increased by 18.4% and 11.1%, respectively. This study is expected to offer significant insights for the widespread adoption of FA while mitigating environmental pollution simultaneously.

## Figures and Tables

**Table 1:** Formulations of SBR/SSBR@FA composites.

Material	Amount/Phr *^a^*
SBR1502	100
Zinc oxide	2.18
Stearic acid	0.73
N-cyclohexylbenzothiazole-2-sulphenamide	1.09
2-Mercaptobenzothiazole	0.73
Sulfur	1.60
SSBR@FA	15/30

*^a^* Parts-per-hundred rubber.

**Table 2:** Mechanical properties of SBR, SBR/FA, and SBR/SSBR@FA vulcanizates.

Samples	Modulus at 100% Elongation/MPa	Modulus at 300% Elongation/MPa	Elongation at Break/%	Tensile Strength/MPa	Tear Strength/KN/m
SBR	0.84 ± 0.01	1.52 ± 0.04	345 ± 12	1.69 ± 0.03	10.4 ± 0.38
SBR/FA-15	1.19 ± 0.06	–	284 ± 3	1.95 ± 0.03	12.9 ± 0.50
SBR/FA-30	1.12 ± 0.05	–	270 ± 2	1.56 ± 0.03	12.4 ± 0.40
SBR/1PFA-15	0.91 ± 0.01	1.62 ± 0.04	387 ± 29	1.93 ± 0.11	14.6 ± 1.09
SBR/1PFA-30	1.04 ± 0.02	1.78 ± 0.01	433 ± 29	2.26 ± 0.15	14.9 ± 0.88
SBR/3PFA-15	0.95 ± 0.02	1.77 ± 0.04	423 ± 31	2.37 ± 0.24	15.1 ± 0.70
SBR/3PFA-30	1.07 ± 0.02	2.15 ± 0.02	406 ± 33	2.81 ± 0.24	15.9 ± 0.23
SBR/1UFA-15	0.96 ± 0.03	1.63 ± 0.06	483 ± 13	2.47 ± 0.19	13.9 ± 1.00
SBR/1UFA-30	1.02 ± 0.02	1.79 ± 0.11	396 ± 28	2.16 ± 0.03	16.7 ± 0.15
SBR/3UFA-15	0.94 ± 0.04	1.85 ± 0.11	419 ± 7	2.44 ± 0.13	16.8 ± 2.64
SBR/3UFA-30	1.06 ± 0.01	2.08 ± 0.09	357 ± 17	2.32 ± 0.19	17.3 ± 0.13

## Data Availability

The original contributions presented in this study are included in the article/Supplementary Materials. Further inquiries can be directed to the corresponding authors.

## Supplementary Materials

The following supporting information can be downloaded at: https://www.mdpi.com/article/10.3390/polym18030348/s1, Figure S1. The dispersion of FA, BFA, 3PFA, and 3UFA in cyclohexane; Figure S2. SEM and EDS mapping diagrams of FA; Figure S3. TEM images of 1PFA and 3UFA; Table S1. The main chemical composition of FA; Table S2. Payne effect in SBR/FA and SBR/SSBR@FA composites; Table S3. Thermal conductivity and flame-retardant properties of SBR and SBR/SSBR@FA composites.
