# Supplementary material for: Preparation of Low-Surface-Energy SSBR@FA Hybrid Fillers via Solution Mechanochemical Approach and Its Enhancement in Mechanical Strength on the Modified FA/SBR Composites
Source: Polymers (Basel). 2026 Jan 28;18(3):348. doi: 10.3390/polym18030348 (PMC12899258; doi:10.3390/polym18030348)
Supplement: Supplementary file 1 [file polymers-18-00348-s001.zip › polymers-4088101-supplementary.pdf]

Preparation of Low-Surface-Energy SSBR@FA Hybrid Fillers via Solution Mechanochemical Approach and Its Enhancement in Mechanical Strength on the Modified FA/SBR Composites

Wei Gao[1,2,*], Jiangshan Zhao[1,2], Wei Qi[2], Zhaohui Huang[1,2,*], Guofeng Liu[2], Chuanqi Feng[1,2], Chao Sang[1,2], Xiujuan Wang[3], Xiaolei Zhang[1,2]

[1] Shandong Provincial Key Laboratory of Monocrystalline Silicon Semiconductor Materials and Technology, Dezhou University, Dezhou 253023, China;
[2] College of Chemistry and Chemical Engineering, Experimental Management Center, Institute of Biophysics, Dezhou University, Dezhou 253023, China;
[3] State Key Laboratory of Advanced Optical Polymer and Manufacturing Technology/Key Laboratory of Advanced Rubber Material, Ministry of Education, Qingdao University of Science and Technology, Qingdao 266042, China;

1. The main chemical composition of FA

Table S1. The main chmeical composition of FA

Samples	SiO_2	CaO	Al_2O_3	SO_3	Fe_2O_3	Other metal oxides
Wt (100%)	31.7	28.7	23.0	5.0	3.6	8.0

2. The dispersion of FA, BFA, 3PFA, and 3UFA in cyclohexane

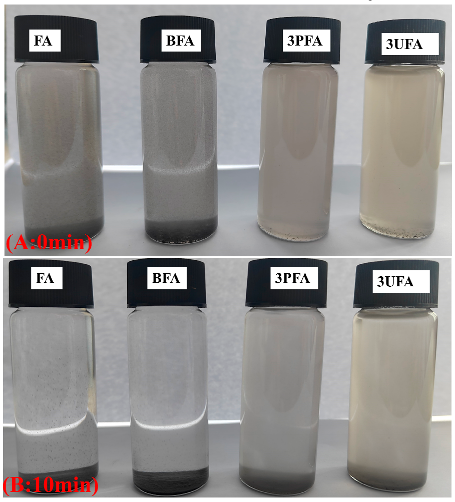

Figure S1. The dispersion of FA, BFA, 3PFA, and 3UFA in cyclohexane: (A) initial state (B) state after ten minutes.

3. SEM and EDS mapping diagrams of FA

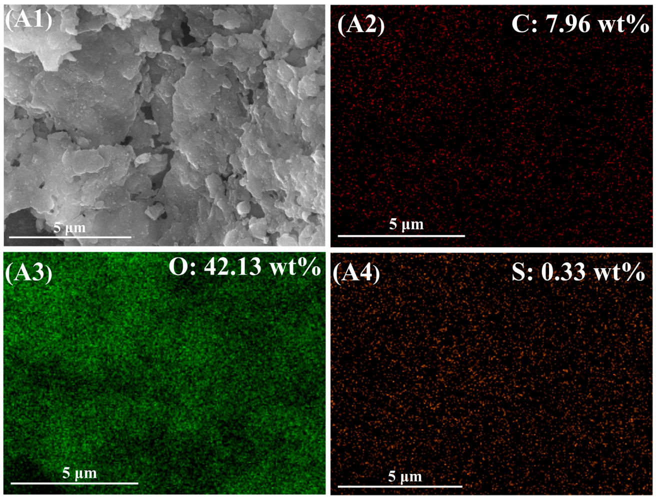

Figure S2. SEM and EDS mapping diagrams of FA.

4. TEM images of 1PFA and 3UFA

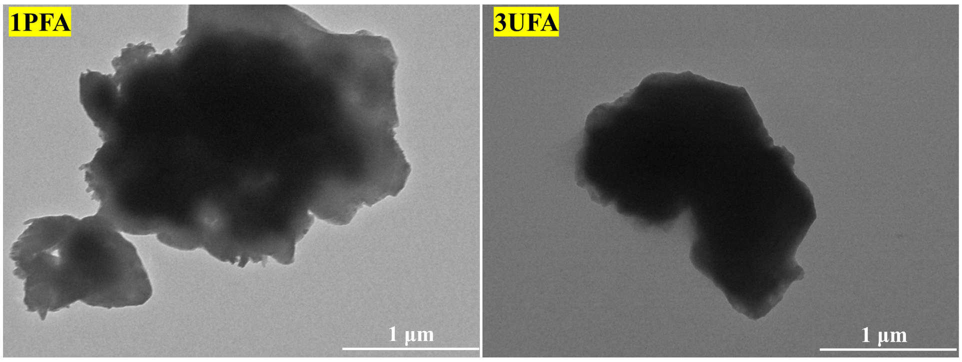

Figure S3. TEM images of 1PFA and 3UFA

5. Payne effect in SBR/FA and SBR/SSBR@FA composites

Table S2. Payne effect in SBR/FA and SBR/SSBR@FA composites

Samples	ΔG' (compounds)[a] (KPa)	ΔG' (vulcanizates)[b] (KPa)
SBR/FA-15	174.65	80.79
SBR/1PFA-15	168.33	45.17
SBR/3PFA-15	167.58	31.95
SBR/1UFA-15	168.31	36.50
SBR/3UFA-15	127.84	55.61
SBR/FA-30	280.31	159.32
SBR/1PFA-30	211.15	87.78
SBR/3PFA-30	204.51	89.87
SBR/1UFA-30	178.94	46.40
SBR/3UFA-30	182.49	62.94

[a]The difference in G' between compounds at 0.28% strain and 200.00% strain.

[b]The difference in G' between vulcanizates at 0.28% strain and 42.00% strain.

6. Thermal conductivity and flame-retardant properties of SBR and SBR/SSBR@FA composites

Table S3. Thermal conductivity and flame-retardant properties of SBR and SBR/SSBR@FA composites

Samples	SBR	SBR/3PFA-15	SBR/3PFA-30	SBR/3UFA-15	SBR/3UFA-30
Thermal conductivity （W/m.k）	0.2293	0.2497	0.2716	0.2443	0.2677
LOI(100%)	19.8	21.5	22.0	21.8	22.5
